# The association between consumption of dairy products and risk of type 2 diabetes

**DOI:** 10.1097/XCE.0000000000000318

**Published:** 2024-12-04

**Authors:** Soroor Fathi, Mahsa Vahdat, Zahra Saeedirad, Naeemeh Hassanpour Ardekanizadeh, Mahdi Mousavi Mele, Soheila Shekari, Khadijeh Abbasi Mobarakeh, Hanieh Shafaei, Alireza Mosavi Jarrahi, Asma Rajabi Harsini, Sara Khoshdooz, Maryam Gholamalizadeh, Hamideh YazdiMoghaddam, Saeid Doaei

**Affiliations:** a Department of Nutrition, Faculty of Allied Medical Sciences, Ahvaz Jundishapur University of Medical Sciences; b Department of Nutrition, School of Allied Medical Sciences, Ahvaz Jundishapur University of Medical Sciences, Ahvaz; c Department of Clinical Nutrition and Dietetics, Faculty of Nutrition and Food Technology, Shahid Beheshti University of Medical Sciences, Tehran; d Department of Clinical Nutrition, School of Nutrition and Food Sciences, Shiraz University of Medical Sciences, Shiraz; e Department of Nutrition, Tabriz University of Medical Sciences, Tabriz; f Department of Nutrition, Science and Research Branch, Islamic Azad University, Tehran; g Department of Community Nutrition, Nutrition and Food Security Research Center, School of Nutrition and Food Science, Isfahan University of Medical Sciences, Isfahan; h Shahid Beheshti College of Midwifery, Guilan University of Medical Sciences, Rasht; i Cancer Research Centre, Shahid Beheshti University of Medical Sciences; j Department of Clinical Nutrition, School of Nutritional Sciences and Dietetics, Tehran University of Medical Sciences, Tehran; k Faculty of Medicine, Guilan University of Medical Sciences, Rasht; l Cancer Research Center, Shahid Beheshti University of Medical Sciences, Tehran; m Operating Room Department, Faculty of Paramedics, Iranian Research Center on Healthy Aging, Sabzevar University of Medical Sciences, Sabzevar; n Department of Community Nutrition, National Nutrition and Food Technology Research Institute, Faculty of Nutrition Sciences and Food Technology, Shahid Beheshti University of Medical Sciences, Tehran; o Reproductive Health Research Center, Department of Obstetrics and Gynecology, School of Medicine, Al-Zahra Hospital, Guilan University of Medical Sciences, Rasht, Iran

**Keywords:** cheese, dairy products, milk, type 2 diabetes, yogurt

## Abstract

**Background:**

The effects of dairy products on type 2 diabetes mellitus (T2DM) are unclear. Some studies have revealed the beneficial effects, whereas others found harmful effects of dairy products on the risk of T2DM. The objective of the present study was to investigate the association of different types of dairy products with risk of T2DM in Iranian adults.

**Methods:**

This cross-sectional study included a total of 4241 individuals. Among these participants, 1804 were diagnosed with T2DM or prediabetes, whereas the remaining 2437 individuals were without T2DM. A validated food frequency questionnaire was used to assess the consumption of different types of dairy products.

**Results:**

A positive association was found between T2DM with dietary intake of milk [odds ratio (OR): 1.16, 95% confidence interval (CI): 1.11–1.23, *P* = 0.008] and cheese (OR: 1.90, 95% CI: 1.41–2.29, *P* = 0.001) after adjustment for age, sex, physical activity, BMI, education level, energy, and fat intake. There was no significant association between T2DM and dietary intake of total dairy, yogurt, ayran (yogurt drink), and curd.

**Conclusion:**

A positive association was found between the consumption of some dairy products including milk and cheese and the risk of T2DM. Further longitudinal studies are warranted to approve this finding.

## Introduction

Diabetes mellitus (DM) is a prevalent pathological disease that often requires the use of oral antidiabetic agents or subcutaneous insulin injections to achieve and sustain glycemic regulation [1]. About 425 million people globally were reported to have DM in 2017, with type 2 diabetes mellitus (T2DM) causing approximately 91% of all cases. By 2045, 629 million more people are expected to have T2DM, and 352 million more individuals with impaired glucose tolerance will be at high risk of developing DM [2]. Certain chronic diseases, such as T2DM, increase the risk of other chronic diseases along with several cardio-metabolic disorders such as obesity, hypertension, and dyslipidemia [3]. Lifestyle interventions, such as increasing physical activity, and adhering to a healthy diet for weight loss, have been shown to be generally effective in reducing insulin resistance and partially restoring pancreatic beta-cell function [4].

Dairy products, rich in calcium, show potential in attenuating inflammation and accumulation of fat, whereas leucine in dairy proteins may counter mitochondrial dysfunction and enhance insulin sensitivity [5,6]. Furthermore, the bioactive lipid trans-palmitoleate (tC16:1n-7) in full-fat dairy is reported to be related to a lower incidence of T2DM, indicating that dairy intake might give protective benefits against T2DM via several mechanisms such as reduced adiposity and better insulin resistance [7,8]. Nonetheless, the high-fat content of certain dairy products (e.g. milk and cheese) can offset the advantages of consuming more calcium or other potentially healthy dairy components [9]. Epidemiological studies have reported conflicting results on the link between T2DM and dairy products. Some studies found a reduced risk associated with consuming more dairy products [10–13], whereas others have found no association [11,14,15]. Although previous studies reported a negative association between the consumption of some dairy products, such as low-fat dairy products [10–12], milk [16], low-fat or skim milk [12], cheese [17], and yogurt [11,16–18], the relationship between different types of dairy products and T2DM in different populations requires further investigation. So, this study aimed to investigate the association between dairy product consumption and the risk of T2DM in Iranian adults.

## Methods

This cross-sectional study was performed using Persian Sabzevar cohort data, which was conducted on 4241 participants, including 742 people with T2DM, 1062 people with prediabetes, and 2437 people without diabetes, and the sampling method was the census. The inclusion criteria included age 35–70 years, interest in participating in the study, completion of the consent form, no contraindication due to following a special diet or allergy, or no prohibition of dairy products. The exclusion criteria also include unwillingness to continue cooperation in the study, being unable to collect the necessary data, calorie intake lower than 500 kcal and higher than 6000 kcal, and current use of calcium and vitamin D supplements. Persian cohort questionnaire data were used on participants’ sociodemographic status. Weight and height were measured with the SECA 755 mechanical scale and the SECA 204 movable stadiometer. The BMI of individuals was calculated as weight in kilograms divided by height in square meters (kg/m^2^).

### Nutritional status and taking dietary supplements

To evaluate the intake of different types of dairy products including milk, yogurt, cheese, ayran, and curd, the Persian cohort food frequency questionnaire (FFQ) was used, and its validity and reliability have already been confirmed [19]. The frequency of food consumption over the past year was examined through a face-to-face interview. Household measures were taken into account for portion sizes and then were converted to grams. The food composition table (FCT) of the United States Department of Agriculture was used to evaluate the amount of energy and macronutrients. The Iranian FCT was considered for local foods that did not exist in the FCT. Data collected from FFQ were converted to grams of nutrients using Nutritionist IV software (First Databank Division, the Hearst Corporation, modified for Iranian foods).

### Biochemical analysis

Blood samples were collected from the participants after an overnight fasting period before the examination, and thereafter their serum was separated and stored at a temperature of −70 °C. Prediabetes was diagnosed as fasting blood sugar (FBS) between 100 and 125 mg/dl (5.6–6.99 mmol/L) and diabetes was diagnosed as FBS higher than 126 mg/dl (≥6.99 mmol/L) according to the American Diabetes Association 2020 criteria [20] or receipt of blood glucose-lowering treatment.

### Data analysis

Descriptive statistics including mean ± SD and number (%) were used to characterize the demographic, social, and anthropometric characteristics of the participants, and logistic regression was used to determine the link between dairy consumption and T2DM after adjusting the effect of confounder variables. Statistical significance was determined by considering a probability level of *P* <0.05 in all computations. The statistical analysis of the results was performed using SPSS software version 22.

## Results

Table 1 displays the characteristics of the participants. The diabetic patients had higher age (54.53 ± 7.75 vs. 48.36 ± 8.62 years, *P* = 0.001), weight (76.18 ± 13.49 vs. 73.77 ± 13.4 kg, *P* = 0.001), BMI (29.24 ± 4.74 vs. 28.02 ± 4.70 kg/m^2^, *P* = 0.001), FBS (169.95 ± 69.2 vs. 96.87 ± 19.39 mg/dl, *P* = 0.001), compared with healthy individuals. The patients with T2DM had lower height (161.4 ± 9.37 vs. 162.33 ± 9.1873 cm, *P* = 0.03), physical activity as the metabolic equivalent for task (37.25 ± 6.86 vs. 38.86 ± 7.91 kcal/kg/h, *P* = 0.001), and last education level (*P* = 0.001) compared with the others.

**Table 1:** Characteristics of the participants

	Diabetic patients	Healthy subjects	*P* value
Age (years)	54.53 ± 7.75	48.36 ± 8.62	0.001
MET (kcal/kg/h)	37.25 ± 6.86	38.86 ± 7.91	0.001
Height (cm)	161.4 ± 9.37	162.33 ± 9.1873	0.03
Weigh (kg)	76.18 ± 13.49	73.77 ± 13.4	0.001
BMI (kg/m^2^)	29.24 ± 4.74	28.02 ± 4.70	0.001
FBS	169.95 ± 69.2	96.87 ± 19.39	0.001
Males (*N*, %)	267 (45.3)	1604 (44.4)	0.68
Last education (*N*, %)			
Diploma	172 (29.2)	817 (22.6)	0.001
Higher education	80 (13.6)	621 (17.2)	
Married (*N*, %)	544 (92.4)	3383 (93.7)	0.61

FBS, fasting blood sugar; MET, metabolic equivalent for task.

The daily consumption of dairy products and macronutrients is indicated in Table 2. The patients with T2DM had a lower intake of energy (2381.79 ± 802.17 vs. 2516.77 ± 780.70 kcal, *P* = 0.001), protein (76.60 ± 25.975 vs. 78.99 ± 26.06 g/day, *P* = 0.04), total lipid (61.47 ± 26.28 vs. 64.99 ± 25.13 g/day, *P* = 0.003), and carbohydrate (393.16 ± 140.01 vs. 415.90 ± 137.51 g/day, *P* = 0.001) compared with the others. Also, the patients with T2DM had a higher dietary intake of cheese (23.34 ± 21.63 521 vs. 21.11 ± 19.54 g/day, *P* = 0.02) compared with the others. There was no significant difference in the mean intake of ayran, curd, milk, and yogurt, and total dairy.

**Table 2:** Dietary intake of dairy products and macronutrients

	Diabetic patients	Healthy subjects	*P* value
Energy (kcal/day)	2381.79 ± 802.17	2516.77 ± 780.70	0.001
Protein (g/day)	76.60 ± 25.975	78.99 ± 26.06	0.04
Total lipid (g/day)	61.47 ± 26.28	64.99 ± 25.13	0.003
Carbohydrate (g/day)	393.16 ± 140.01	415.90 ± 137.51	0.001
Milk (g/day)	73.34 ± 103.75	65.53 ± 89.57	0.08
Yogurt (g/day)	123.96 ± 89.34	124.94 ± 97.55	0.81
Cheese (g/day)	23.34 ± 21.63 521	21.11 ± 19.54	0.02
Ayran (g/day)	88.47 ± 103.00	90.72 ± 109.47	0.62
Curd (g/day)	6.65 ± 8.93	6.30 ± 10.76	0.40
Total dairy (servings/day)	316.22 ± 196.57	308.77 ± 194.68	0.39

**Fig. 1:**
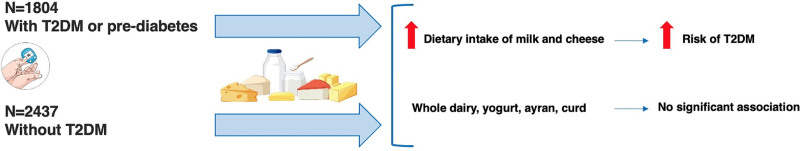
The association between consumption of dairy products and risk of T2DM. T2DM, type 2 diabetes mellitus.

The association between different dairy products and the risk of T2DM is presented in Table 3. A positive association was found between T2DM and dietary intake of milk [odds ratio (OR): 1.16, 95% confidence interval (CI): 1.11–1.23, *P* = 0.008] and cheese (OR: 1.90, 95% CI: 1.41–2.29, *P* = 0.001) after adjustment for age, sex, education level, physical activity, BMI, and energy and fat intake. These results indicated that each g increase in the daily intake of milk and cheese was significantly associated with a 16 and 90% increase in the odds of T2DM. There was no significant association between dietary intake of whole dairy, yogurt, ayran, curd, and T2DM (Fig. 1).

**Table 3:** Logistic regression of the association between dairy products and diabetes

	Milk		Yogurt		Cheese		Ayran		Curd	
	OR (95% CI)	*P* value	OR (95% CI)	*P* value	OR (95% CI)	*P* value	OR (95% CI)	*P* value	OR (95% CI)	*P* value
Model 1	1.001 (1.00–1.002)	0.061	1.00 (0.99–1.001)	0.520	1.50 (1.10–1.90)	0.012	1.00 (0.99–1.01)	0.551	1.003 (0.99–1.01)	0.524
Model 2	1.01 (1.00–1.02)	0.061	1.00 (0.99–1.001)	0.381	1.70 (1.20–2.10)	0.002	1.00 (1.00–1.01)	0.371	1.001 (0.99–1.009)	0.791
Model 3	1.15 (1.14–1.23)	0.020	1.00 (0.99–1.001)	0.441	1.70 (1.20–2.10)	0.002	1.00 (0.99–1.01)	0.733	1.002 (0.99–1.01)	0.633
Model 4	1.16 (1.11–1.23)	0.008	1.00 (0.99–1.001)	0.910	1.90 (1.41–2.29)	0.001	1.00 (1.00–1.01)	0.312	1.004 (0.99–1.01)	0.352

Model 1: crude; model 2: adjusted for age and sex; model 3: additional adjustment for education level, physical activity, and BMI; and model 4: additionally adjusted for energy and fat intake.

CI, confidence interval; OR, odds ratio.

## Discussion

A positive association was found between dietary intake of milk and cheese and the risk of T2DM. This association remained significant even after adjusting for other important T2DM risk factors. The observed discrepancy between the results of the present study and previous investigations may be attributed to the traditional Iranian food habits, such as low intake of milk, more intake of foods like bread, or to the variety of milks and cheeses consumed in Iran.

Some previous studies reported a reduced risk of T2DM with moderate consumption of dairy products such as low-fat dairy products, cheese, and yogurt [21,22]. In addition, a previous cohort study indicated a negative association between intake of low-fat dairy products, milk, and/or yogurt and the risk of T2DM. Also, daily consumption of low-fat dairy products was associated with a 12% decrease in the risk of T2DM [15]. Furthermore, higher consumption of milk, yogurt, and total dairy products was associated with a lower risk of T2DM over a 4-year follow-up in a Chinese cohort study [16]. A meta-analysis of 15 studies indicated that a higher intake of fermented dairy foods, particularly yogurt, is associated with a significantly decreased risk of DM [18]. Daily consumption of vitamin D from dairy products may increase circulating leptin and ghrelin and ultimately lead to improved insulin sensitivity and, thus, the regulation of beneficial appetite hormones [21,23].

On the other hand, some other studies found no association between dairy consumption and T2DM incidence [24,25]. Also, another study reported that full-fat dairy products (whole milk, cream, sour cream, cream cheese, and other cheeses) were not associated with T2DM risk [26]. In a prospective cohort study investigating Dutch adults aged 55 and older, no significant associations were found between various dairy products including total, skimmed, semiskimmed, full-fat, fermented, and nonfermented dairy, and the prevalence of T2DM during a 9.5-year follow-up [27].

The higher saturated fat content of dairy products may counteract the beneficial effects of dairy and increase the risk of T2DM through increased insulin resistance [28,29]. The fat composition of dairy products is directly related to adverse changes in plasma lipoprotein levels [30]. Replacing saturated fatty acids with unsaturated fatty acids reduced not only LDL cholesterol but also VLDL and triglycerides [31]. This adverse effect of dairy products on the risk of DM, which was found in the present study may be due to the high concentration of saturated fat and natural trans-fat in the circulation and tissue of ruminants, which is associated with a lower risk of DM [32]. However, data on the amount of fat in dairy products consumed by the participants was not available in the present study. Moreover, different studies reported consumption of different dairy products, different preferred types of dairy products, and different ranges of their intakes, which may partly explain the contradictory findings between the studies [33]. For example, as confirmed in this study, the annual consumption of milk in Iran is much lower than the mean consumption worldwide (75 kg/capita vs. 300–350 kg/capita) [34].

In our study, there was no significant difference between the consumption of fermented dairy products (yogurt, ayran, and curd). Although previous research has shown that consuming fermented dairy products, particularly yogurt, is associated with a lower risk of T2DM [23,32]. This difference in results may be due to the dose-response characteristics of dairy products [35], which can be influenced by the amount of consumption of dairy products by the participants. Moreover, recent studies indicated that the difference in the pattern of bacterial strains in fermented products may be involved in their effect on health outcomes such as DM [36].

However, the present study is accompanied by certain limitations, most notably the utilization of a questionnaire to collect dietary data, which introduces the potential for inherent biases and inaccuracies associated with self-reported information. Furthermore, the cross-sectional design of this study limits our ability to establish a causal relationship between dairy product consumption and risk factors for T2DM. Because the data are collected at a single point in time, preventing us from analyzing temporal associations or causal pathways that may develop over time. Also, data on different types of low-fat and high-fat dairy products as well as fermented and non-fermented dairy products was not available. To increase our understanding of the relationship between different types of dairy product consumption and T2DM, additional research is required.

### Conclusion

In summary, this study provided some evidence that milk and cheese may have adverse effects on the risk of T2DM. Confirmation of our study’s results by future dose-response studies could significantly influence nutrition recommendations for those with T2DM. To more understanding, further longitudinal studies are suggested to explore mechanisms, diverse populations, and the impact of different dairy products on T2DM risk. This comprehensive approach will inform more precise dietary recommendations for managing T2DM.

## Acknowledgements

The authors extend their gratitude to all the participants who willingly engaged in this investigation. The present manuscript was obtained through an authorized research project at Sabzevar University of Medical Sciences in Sabzevar, Iran.

The research was funded by Sabzevar University of Medical Sciences, Sabzevar, Iran (Code 99218).

S.F., M.V., Z.S., N.H.A., M.M., S.S., and S.D. designed the study and were involved in the data collection, analysis, and drafting of the manuscript. H.S., K.A.M., A.M.J., A.R.H., S.K., M.G., H.Y., and S.D. were involved in the design of the study, analysis of the data, and critically reviewed the manuscript. All authors read and approved the final manuscript.

The Ethical Committee of the Research Ethics Committee at Sabzevar University of Medical Sciences in Sabzevar, Iran, provided approval for this study (code: IR.MEDSAB.REC.1400.040). Every experiment involving human subjects was carried out in compliance with the 1964 Declaration of Helsinki, any updates thereto, ethical guidelines established by the applicable national or institutional research council, or equivalent standards of ethics. Written informed permission was acquired by each subject.

The datasets used and/or examined in the present investigation can potentially obtained from the corresponding author upon reasonable request.

### Conflicts of interest

There are no conflicts of interest.
